# Decreased Mitochondrial Membrane Potential Activates the Mitochondrial Unfolded Protein Response

**DOI:** 10.17912/micropub.biology.000445

**Published:** 2021-09-10

**Authors:** Brandon J. Berry, Tyrone O. Nieves, Andrew P. Wojtovich

**Affiliations:** 1 University of Rochester Medical Center, Department of Anesthesiology and Perioperative Medicine, 575 Elmwood Ave., Rochester NY, 14642 Box 711/604.

## Abstract

Mitochondria are ATP-producing organelles that also signal throughout the cell. Mitochondrial protein homeostasis is regulated through membrane potential-dependent protein import and quality control signaling. The mitochondrial unfolded protein response (UPR^mt^) is a specific program that responds to imbalances in nuclear and mitochondrial gene expression. Mounting evidence suggests that the electrochemical gradient that powers mitochondrial function, the mitochondrial membrane potential (Δψ_m_), is a core regulator of the UPR^mt^. Here we tested this notion directly by pharmacologically dissipating Δψ_m _and monitoring UPR^mt ^activation. We found that chemical dissipation of Δψ_m _using FCCP indeed activated UPR^mt^ dose-dependently in *C. elegans *assayed by the HSP-60::GFP reporter strain.

**Figure 1. FCCP dose-dependently activates the mitochondrial unfolded protein response (UPRmt):**
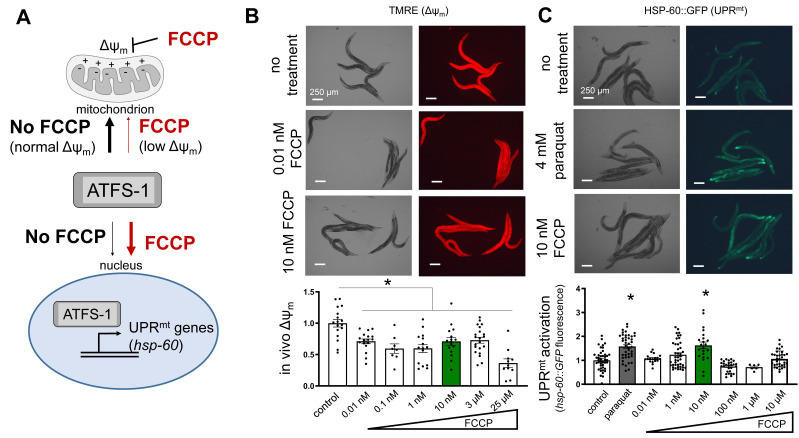
A) Schematic depicting transcriptional control of the UPR^mt^. The UPR^mt ^transcription factor (ATFS-1 in *C. elegans*) is imported to mitochondria according to the mitochondrial membrane potential (Δψ_m_) and degraded. Δψ_m_ is denoted by “+” and “–” across the mitochondrial inner membrane. When the transcription factor enters the nucleus target genes such as *hsp-60* are transcribed to respond to mitochondrial stress. FCCP is a protonophore that shuttles protons across the mitochondrial inner membrane resulting in decreased Δψ_m. _B) Adult *C. elegans* are shown after treatment with TMRE, a potentiometric Δψ_m _indicator. Quantification of fluorescence is shown below the representative images. From left to right, each bar represents the mean fluorescence from N = 17, 17, 8, 15, 16, 21, 11 animals. C) Adult *C. elegans* are shown expressing HSP-60::GFP. Control is compared to paraquat, a known UPR^mt^ activator, and to FCCP treatment. Quantification is shown below the representative images. From left to right, each bar represents the mean fluorescence from N = 44, 42, 18, 42, 22, 28, 6, 33 animals. Raw fluorescence values are shown normalized to no treatment control in all cases. Scale bar is 250 μm in all images. Experimental N values are individual animals. Data were obtained across at least 3 different days. *p < 0.05 compared to control by one-way ANOVA with Bonferroni’s test in all cases. Data are means + SEM.

## Description

Mitochondria are organelles that make ATP by generating a proton gradient across the inner membrane through the action of the electron transport chain (ETC). The gradient is composed of a membrane potential (Δψ_m_) and a concentration gradient (ΔpH). In addition to energy production, mitochondria are recognized as signaling organelles (Chandel 2015), and Δψ_m _powers diverse signaling outputs that mitochondria coordinate. One such signaling response is the mitochondrial unfolded protein response (UPR^mt^). Some ETC complex components are encoded by mitochondrial or nuclear genes and require coordination between the genomes for proper stoichiometry (Shpilka and Haynes 2018). The UPR^mt^ is a protein homeostasis response that is activated when nuclear and mitochondrial gene expression are not correctly coordinated (Melber and Haynes 2018). When the expression of mitochondrial and nuclear encoded proteins is mismatched, the UPR^mt ^is activated. In *C. elegans*, the UPR^mt ^is controlled by the transcription factor ATFS-1, which is normally trafficked to mitochondria and degraded. When nuclear:mitochondrial gene expression is perturbed, ATFS-1 is blocked from entering mitochondria and is instead trafficked to the nucleus where a host of chaperones and stress-response genes are activated and transcribed.

Recently, it was suggested that ATFS-1 entry to mitochondria for degradation depended on sufficient Δψ_m_ (Rolland *et al.* 2019). *In vivo* evidence supported this assertion, and implied that Δψ_m_ is a main regulator of mitochondrial protein homeostasis through its role in driving ATFS-1 import to mitochondria (Poveda-Huertes *et al.* 2021; Shpilka *et al.* 2021). Based on this premise, we sought to directly test if targeting Δψ_m _could alter UPR^mt ^signaling. We predicted that decreased Δψ_m _would result in activation of UPR^mt^ due to decreased driving force for ATFS-1 to enter mitochondria and be degraded. This would lead to ATFS-1 localization to the nucleus and activation of the UPR^mt ^target gene *hsp-60* (**Figure 1A**). We used the protonophore FCCP at various doses to decrease Δψ_m _in live *C. elegans.* Δψ_m_ was monitored in live, adult worms using the potentiometric fluorescent indicator, TMRE.At all doses tested Δψ_m _was decreased compared to untreated control (**Figure 1B**). Using a similar dose range, we found that one intermediate dose was sufficient to activate UPR^mt ^signaling, assayed through the characterized transcriptional *hsp-60* reporter strain (Melber and Haynes 2018; Rolland *et al.* 2019) (Figure 1C). Paraquat, a known UPR^mt ^positive control (Kim and Sieburth 2018), was used to confirm our results with FCCP. This study was repeated on at least 3 independent days.

The UPR^mt ^is linked to many stress-resistance signaling responses, including hypoxia resistance (Peña *et al.* 2016). We previously showed that FCCP protected against hypoxia (Berry *et al.* 2020b), and this work suggests that UPR^mt ^may have been involved. Our other work, however, implicated energy-sensing signaling downstream of decreased Δψ_m _and hypoxia resistance (Berry *et al.* 2020a). The requirement of UPR^mt^ for hypoxia resistance downstream of decreased Δψ_m _remains untested. Further, the UPR^mt ^has been implicated in many disease models that are not fully characterized (Jovaisaite *et al.* 2014). It is likely that many changes occur with decreased Δψ_m_, and it will be necessary to characterize changes in Δψ_m _and other mitochondrial functions in a wide range of contexts to uncover molecular mechanisms of mitochondrial signaling. For example, it is unclear why only a certain dose (10 nM) of FCCP activated the UPR^mt ^here when all doses resulted in mitochondrial depolarization. This result suggests that other cellular mechanisms likely control UPR^mt^ activation in addition to Δψ_m_. In addition, the level of mitochondrial depolarization may differentially result in UPR^mt ^or mitochondrial autophagy, a process that is also responsive to decreased Δψ_m_. The mechanism that controls which cellular response is elicited likely depends on the degree of mitochondrial stress. This work is complementary to the established role of mitochondrial ETC stress and UPR^mt^. Inhibition of the ETC results in activation of UPR^mt^, but only if initiated before the L3/L4 stage in *C. elegans* (Durieux *et al.* 2011). Our results suggest that the mechanism of ETC mediated UPR^mt ^activation may be somewhat distinct from the Δψ_m _control over UPR^mt^, because decreased Δψ_m _was able to induce UPR^mt ^after the L3/L4 stage.

## Methods

*Fluorescence imaging. C. elegans* were fed OP50 bacteria on nematode growth media (NGM) plates. L4 animals were synchronized by egg lay and were stained by exposure to 100 nM TMRE for 24 hours that was applied to plates (Cho *et al.* 2017; Berry *et al.* 2020B). Fluorescence was photographed in day 1 adult animals on 2% agarose pads with tetramisole (0.1% w/v) for anesthetic. Red TMRE fluorescence was captured using TexasRed filter sets, and green GFP was captured using GFP filter sets on an epifluorescence microscope (Olympus MVX) equipped with a Lumenera camera and acquisition software (Infinity Analyze; Lumenera). Fluorescence of individual animals was quantified using ImageJ. ROIs were whole animals, and background was manually subtracted from final measurements in ImageJ. Mean intensity was measured, and after background subtraction, was normalized to control intensity. Paraquat was dissolved in water and applied to plates at a final concentration of 4 mM. FCCP was dissolved in ethanol (< 0.1% final concentration, consistent across all concentrations) and applied to plates at various concentrations (see figure). Animals were exposed to paraquat or FCCP for 24 hours.

## Reagents

**Table:** 

Strain	Genotype	Source
N2	wildtype	CGC
SJ4058	zcIs9 [*hsp-60*::GFP + *lin-15*(+)] V	CGC
